# Associated oral manifestations with HIV southeastern Brazilian patients on antiretroviral therapy

**DOI:** 10.1016/j.bjorl.2023.01.001

**Published:** 2023-01-23

**Authors:** Brisa Ketrine Lustosa de Souza, Daniele Sorgatto Faé, Cleidiel Aparecido Araújo Lemos, Francielle Silvestre Verner, Renato Assis Machado, Rose Mara Ortega, Sibele Nascimento de Aquino

**Affiliations:** a Universidade Federal de Juiz de Fora (UFJF-GV), Departamento de Odontologia, Governador Valadares, MG, Brazil; b Universidade Estadual de Campinas, Faculdade de Odontologia de Piracicaba, Departamento de Diagnóstico Bucal, Piracicaba, SP, Brazil

**Keywords:** HIV, Antiretroviral therapy highly active, Oral manifestations

## Abstract

**Objectives:**

This study aimed to assess the prevalence of oral lesions in patients living with HIV infection and their association with CD4 count, viral load, and antiretroviral therapy in patients with HIV.

**Methods:**

A cross-sectional study was conducted on a sample of 161 patients attending the… All the patients were examined for their oral lesions, current CD4 counts, type, and duration of the therapy. Data analyses were carried out using Chi-Square, Student T/Mann-Whitney, and logistic regression tests.

**Results:**

Oral lesions were observed in 58.39% of patients with HIV. Periodontal disease with 78 (48.45%) or without mobility 79 (49.07%) was observed more frequently, followed by hyperpigmentation of oral mucosa 23 (14.29%), Linear Gingival Erythema (LGE) 15 (9.32%), candidiasis pseudomembranous 14 (8.70%). Oral Hairy Leukoplakia (OHL) was observed only in 3 (1.86%). A relationship between periodontal disease with dental mobility and smoking was found (*p* = 0.04), as well duration of treatment (*p* = 1.53e-3) and age (*p* = 0.02). Hyperpigmentation was related to race (*p* = 0.01) and smoking (*p* = 1.30e-6). CD4 count, CD4:CD8 ratio, viral load, or type of treatment were not associated with oral lesions. Logistic regression showed that the duration of treatment has a protective effect on the periodontal disease with dental mobility (OR = 0.28 [−2.27 to −0.25]; *p*-value = 0.03), independent of age or smoking. To hyperpigmentation, the best model included smoking (OR = 8.47 [1.18–3.10], *p* =  1.31e-5), without race or type and duration of treatment.

**Conclusion:**

Among HIV patients undergoing antiretroviral treatment, oral lesions can be observed, predominantly periodontal disease. Pseudomembranous candidiasis and oral hairy leukoplakia were also observed. No relationship was found between associated oral manifestations in HIV patients and the start of the treatment, TCD4+ and TCD8+ cell count, TCD4:TCD8 ratio, or viral load. The data indicate that there is a protective effect of duration of treatment with relation to periodontal disease with mobility and that hyperpigmentation seems to be more related to smoking than type and duration of treatment.

**Level of evidence:**

Level 3 (OCEBM Levels of Evidence Working Group*. "The Oxford 2011 Levels of Evidence").

## Highlights

• The associated oral manifestations in HIV patients remain present even in the HAART era.
• Periodontal disease with or without mobility were the predominant oral lesions in the patients of this study.
• No relationship was found between oral lesions, TCD4+ and TCD8+ cell count, TCD4:TCD8 ratio, or viral load.
• There is a protective effect of duration of treatment with relation to periodontal disease with mobility.

## Introduction

Despite all advances in the prevention and treatment, the pandemic of Human Immunodeficiency Virus (HIV) is still a worldwide health problem. Approximately 38 million people globally are living with HIV, being that 1.7 million people acquired HIV and 690.000 lives were lost to AIDS (acquired immunodeficiency syndrome) related illnesses in 2019. Despite the availability of effective treatments, only 67% of people living with HIV have access to Antiretroviral Therapy (ART)^1^. HIV infection results in a reduction in CD4+ T lymphocyte counts, interfering in the immune system leading to greater susceptibility of opportunistic infections and neoplastic processes^2^, ^3^.

Oral lesions are one of the most important indicators of HIV infection^4^, ^5^, ^6^, ^7^. The presence of oral lesions in HIV+ patients is correlated with high viral load and low CD4+ cell counts^8^, ^9^. Some studies have reported differences in the prevalence of oral manifestations associated with HIV infection^5^, ^7^, ^10^, ^11^. The prevalence of oral lesions could be influenced by the development of the country, demographic locality, healthcare system, medicine availability, HIV strains, stage of HIV, deleterious habits, and gender^3^, ^12^, ^13^.

Oral lesions associated with HIV infection have a negative impact on the quality of life of infected patients and make it difficult to treat various systemic diseases^14^. The advent of Antiretroviral Therapy (ART) for the treatment of HIV-infected patients contributed to the reduction of oral lesions and HIV-associated opportunistic infections improving the oral health quality of life^15^, ^16^. Nowadays, the most common combination therapy is known as Highly Active Antiretroviral Therapy (HAART), which is a cocktail therapy combining three or more antiretroviral drugs that act on different targets and it is a standard HIV/AIDS treatment method^7^.

The treatment of HIV infection with HAART increased survival and reduced mortality for AIDS-related diseases^17^. Furthermore, it has been reported a decrease of 10%‒50% of associated oral manifestations with HIV patients^10^. However, even with the significant decrease in the prevalence of HIV-related oral lesions with the systemic use of HAART^10^, ^13^, they continue to be debilitating for HIV-positive patients mainly in low-income countries^12^. Therefore, due to the changes in the conduction of ART, the exploration of main oral lesions, clinical and laboratory profiles of HIV-infected patients should be considered to help the clinicians with the oral health management to identify some common oral characteristics of patients living with HIV and contribute the reduction of the stigma of these peoples^9^. Thus, the study aimed to assess the prevalence of oral lesions in patients living with HIV infection and their association with CD4 count, viral load, and antiretroviral therapy. Also, this study shows the clinical and laboratory data observed in HIV patients during antiretroviral therapy.

## Methods

The ‘Strengthening the Reporting of Observational Studies in Epidemiology (STROBE)’ guidelines^18^ were followed when composing this manuscript.

This cross-sectional study was conducted on a sample of 161 patients attending the Centro de Referência em Atenção Especial à Saúde (CRASE) in Governador Valadares, Brazil. Sample size calculation was realized using the effect size of 0.30, α error of 0.05, power of the sample of 0.80, and significance level <0.05 in. G power software version 3.1.9.6. The patients were registered to the CRASE for their primary health care and regular checkup from July 26 to December of 2019. The study protocol was approved by the research committee at the Federal University of Juiz de Fora, Brazil, under the number (#1.821.072). All information about the patients and their identity was anonymous. Subjects were given both verbal and written information about the nature of the study and written consent was obtained.

The inclusion criteria were patients who were diagnosed as HIV positive and were on ART therapy independent of age and sex and patients diagnosed as HIV positive with viral load indetectable who were not using ART therapy. All adult patients living with HIV/AIDS (PLWHA) who attended the clinic and satisfied the inclusion criteria were recruited. The exclusion criteria were patients who did not follow up the treatment and patients who did not accept to participate in the study.

A well-structured questionnaire to collect relevant social and demographic details from the study patients was applied. The initial or baseline CD4 count, and other data were retrieved from medical records and other relevant documentation. Patient records were evaluated, and oral examination was conducted by a single examiner trained in oral diagnosis (SNA) and performed on all PLWHA at the time of routine medical consultation. A thorough clinical examination of the oral cavity was performed with sterile gauze and spatula under artificial light. Oral lesions were diagnosed according to the criteria of EEC Clearinghouse Classification^19^. Periodontal status was evaluated according to the presence of gingivitis and clinical mobility and classified as a periodontal disease with and/or without mobility.

All the patients were examined for their oral manifestations, current CD4 counts, and the type and duration of the therapy. PLWHA who were found to have dental lesions, such as caries, periodontal disease, or missing teeth, were referred to dental clinics for management.

The subjects were grouped according to the status of CD4 count, viral load type, and duration of treatment received as follows: The CD4 counts which were recorded were divided into under three ranges:1) CD4 count <200; 2) CD4 count between 200 and 500 and 3) CD4 count >500. The viral load (cp/mL of blood) was divided between >1000, 40 to 1000, and undetectable. The CD4+ T cell count (cells/mm^3^) and viral load were considered only when evaluated at most two weeks before data collection. The duration of the HAART therapy was also divided into two ranges: 1) ART therapy ≤2 years and 2) ART therapy >2 years. Type of therapy was classified into monotherapy, dual therapy, and HAART. The star of treatment after diagnosis was divided into ≤1 and >1 year.

All the ranges were expressed in percentages. All the findings were tabulated, and the results were analyzed using measures of central tendency. The data collected were analyzed using JASP Team (2020) software version 0.14.1 (University of Amsterdam). Differences in the prevalence of oral manifestations associated with HIV-infected patients were compared between age, gender, ethnicity, the habits of smoking, alcohol drugs habits, possible transmission, duration of treatment, the start of treatment, antiretroviral therapy, TCD4 cell count, and viral load. Chi-Square, Student *t*, or Mann-Whitney test (according to normality) were applied to assess comparisons between groups. Logistic regression analysis was also realized to evaluate the periodontal disease, smoking, age, and duration of and antiretroviral therapy. As well, hyperpigmentation and duration of treatment and race. The significance level was set at 95% (*p* <  0.05).

## Results

A total of 161 patients with HIV were enrolled in the study. 15 of 176 patients were excluded due to undetectable viral load and absence of treatment at the time of the data collection.

The mean age of HIV patients was 43.63 ± 12.66 years (range: 18–75 years), with a predominance of male 83 (51.55%) than female 78 (48.45%) patients. The mean age at the time of diagnosis was 35.83 ± 11.77 years. Low education (<8 years) was observed in most participants (64%). Regarding the habits of patients, most of the patients reported the non-use of cigarette smoking 121 (75.16%), non-alcohol consumption 106 (65.84%), and illicit drugs 153 (95%) (Table 1). Most of the patients (85.09%) were presumably infected with HIV by the sexual way. About 36% were diagnosed after hospital admission and 135 (83.85%) were symptomatic at the time of diagnosis. The mean of age of diagnosis was 35.83 years ± 11.77 years. The mean of the duration of HIV infection and treatment was 8 years. HAART was used by 105 (65.22%) participants, followed by dual therapy 52 (32.30) and monotherapy 4 (2.48%).

**Table 1:** Presence and absence of oral manifestations in HIV patients on antiretroviral therapy.

Variables	Oral manifestations		*p*-value^a^
	Presence, *n* (%)	Absence, *n* (%)	
Gender			
Female	41 (43.62)	37 (55.22)	0.15
Male	53 (56.38)	30 (44.78)	
Race/ethnicity			
Caucasian	33 (35.48)	26 (38.81)	0.67
Non-Caucasian	60 (64.52)	41 (61.19)	
Uninformed	1 (0.62)		
Cigarette smoking			
Yes	27 (28.72)	13 (19.40)	0.18
No	67 (71.28)	54 (80.60)	
Alcohol consumption			
Yes	34 (36.17)	21 (31.34)	0.61
No	60 (63.83)	46 (68.66)	
Illicit drugs			
Yes	5 (5.32)	3 (4.48)	0.81
No	89 (94.68)	64 (95.52)	
Duration of HIV treatment			
≤2 years	15 (17.05)	17 (25.76)	0.19
>2 years	73 (82.95)	49 (74.24)	
Uninformed	7 (4.34)		
Start of treatment			
≤1 year	85 (90.43)	61 (91.04)	0.89
>1 year	9 (9.57)	6 (8.96)	
TCD4+ cell count^b^			
>200	8 (19.05)	5 (16.13)	0.94
200‒500	20 (47.62)	16 (51.61)	
>500	14 (33.33)	10 (32.26)	
TCD4+: TCD8+ ratio^c^			
<1	35 (87.50)	27 (87.10)	0.96
≥1	5 (12.50)	4 (12.90)	
Viral load‡			
>1000	12 (17.14)	11 (19.64)	0.28
40‒1000	15 (21.43)	6 (10.71)	
Undetectable	43 (61.43)	39 (69.64)	

HAART, Highly Active Anti-Retroviral Therapy.

‡ Data were available from 126 individuals (cp/mL of blood).

a p-values refer to ≤0.05 by Chi-Square test.

b Data available from 73 individuals (cells/mm^3^).

c Data available from 71 individuals.

Associated oral manifestations were observed in 94 patients (58.39%), being 53 (56.38%) and 41 (43.62%) of male and female patients, respectively. Regarding the habits of patients, most of the patients with oral lesions reported the non-use of cigarette smoking 67 (71.28%), non-alcohol consumption 60 (63.83%), and non-use of illicit drugs 89 (94%). A higher prevalence of associated oral manifestations was observed for non-Caucasian patients 60 (64.52%). Among patients with associated oral manifestations, the HAART therapy was considered by most patients 60 (63.83%), followed by dual therapy 31 (32.98%) and monotherapy 3 (3.19%). No differences were observed for the different ART therapies (*p* =  0.76). No statistical difference was observed between sex, race, habits, duration of infection, duration of HIV treatment, and time between diagnosis and start of treatment (Table 1).

No statistical difference was observed between the presence of associated oral manifestations and age (*p* =  0.61) and age at diagnosis (*p* =  0.40).

The CD4+ T cell count (cells/mm^3^) data was available only from 73 patients. The minority of patients 13 (17.81%) had a CD4+ T cell lower than 200. Thirty-six patients had a CD4+ T cell count between 200–500 (49.32%), and 24 (32.88%) had a CD4+ T cell count above 500. Regarding the ratio between CD4+ T and CD8+ T the high prevalence of patients and associated oral manifestations was observed for ratio <1 (87.5%). The viral load (cp/mL of blood) was available from 126 individuals. Associated oral manifestations were observed more frequently on patients with undetectable viral load, however without statistical significance (*p* =  0.28) (Table 1).

This study found the prevalence of 6 associated oral manifestations (Table 2). Between the different oral lesions, a high prevalence of periodontal disease with 78 (48.45%) or without mobility 79 (49.07%) in the HIV-infected patients were observed, followed by hyperpigmentation of oral mucosa 23 (14.29%), Linear Gingival Erythema (LGE) 15 (9.32%), candidiasis pseudomembranous 14 (8.70%). Oral Hairy Leukoplakia (OHL) was observed only in 3 (1.86%). A relationship between smoking and periodontal disease with dental mobility was found (*p* =  0.04), duration of treatment (*p* =  1.53e-3), and with age (*p* =  0.02) but not type of treatment, viral load, or CD4+ T cell count. Hyperpigmentation was related to race (*p* =  0.01) and smoking (*p* =  1.30e-6) but not with type or duration of treatment as well viral load or CD4+ T cell count.

**Table 2:** Oral manifestation in HIV-infected patients during treatment by the duration of HIV infection, start and duration of treatment, antiretroviral therapy, and laboratory data.

	PDM	PD	Hyperpigmentation	Linear gingival erythema	Candidiasis Pseudomembranous	Oral hairy leukoplakia
Smoking						
Yes	25^a^	25	15^a^	3	5	1
No	53	54	8	12	9	2
Race						
Caucasian	26	26	3	8	4	1
Non-Caucasian	51	52	19^b^	7	10	2
Duration of HIV treatment						
≤2 years	7^a^	7^a^	2	6		1
>2 years	65	66	20	8		2
Start of treatment						
≤2 years	70	71	19	‒		3
>2 years	8	8	4	15		‒
Antiretroviral therapy						
Monotherapy	3	3	1	‒	1	‒
Dual Therapy	27	28	10	5	4	1
HAART	48	48	12	10	9	2
TCD4 cell count*						
>500	5	5	4	2	2	1
200‒500	17	18	3	3	2	–
<200	11	11	3	3	2	–
TCD4:TCD8 ratio^c^						
<1	26	27	6	8	4	1
≥1	5	5	2	‒	1	‒
Viral load‡						
>1000	10	10	3	2	2	1
40‒1000	10	10	2	3	1	‒
Undetectable	37	38	11	6	7	‒

PDM, Periodontal Disease with Mobility; PD, Periodontal Disease without mobility; HAART, Highly Active Antiretroviral Therapy.

‡ Data available from 126 individuals (cp/mL of blood).

b Data available from 73 individuals (cells/mm^3^).

a *p*-values refer to ≤0.05 by Chi-Square test.

c Data available from 71 individuals.

According to periodontal disease with mobility, the best model in the logistic regression included duration of treatment, age, and smoking (*r*² = 0.15, *p* =  1.03e-3). The results showed that duration of treatment has a protective effect on periodontal disease with dental mobility (OR = 0.30 [−2.23 to −0.19]; *p*-value = 0.02) and smoking has a risk effect (OR = 2.40 [0.07–1.69]; *p*-value = 0.03). To hyperpigmentation, the best model (r² = 0.21, *p* =  6.93e-6) included only smoking (OR = 8.47 [1.18–3.10], *p* =  1.31e-5).

## Discussion

Oral lesions are the first indicators of HIV infection and progression to AIDS. Although studies suggested that the introduction of new antiretroviral therapies contributes to the decrease of oral lesions^7^, ^11^, ^14^, ^16^^,^^20^, ^21^, ^22^, ^23^, ^24^, the data of this study verified that the associated oral manifestations with HIV patients remain present even in the HAART era. However, it must be observed that ART therapy (mono, dual, and HAART) contributed to a decrease of oral lesions, independently of therapy. These results should be attributed to the fact that the ART therapy contributes to improving the immune status, and consequently the tendency for reduction of oral lesions^25^. Due to the introduction and easier access to ART treatment (mono, dual, and HAART), HIV-infected patients are having a better prognosis and longer life expectancy, and more patients are looking for treatment for their HIV-associated oral lesions than in the past^13^.

HIV-associated oral manifestations have been reported to appear more frequently when there is a decreased TCD4+ cell count and increased viral load^4^, ^9^, ^11^, ^14^. However, these data present contrasts with our results, which showed similar results of oral lesions according to different TCD4+ counts and viral load. It must be important to emphasize that a limited number of patients (*n* = 13) showed TCD4+ below 200 cells/mm^3^ and most patients showed undetectable viral loads. Therefore, these results should be interpreted with caution due to this limitation.

Oral lesions in HIV-infected patients did not follow a common pattern and differs according to regions of the world. Periodontal health should be considered an important factor for a better quality of life in patients living with HIV^26^. Periodontal disease with or without mobility were the predominant oral lesions in the patients of this study. Even in patients with HAART therapy, periodontal disease was also observed, which is a high value compared to other similar studies^7^, ^12^, ^21^, ^27^^,^^28^. The results also indicate a relation between age, smoking, and duration of treatment with periodontal disease with mobility. Logistic regression showed that the duration of treatment is protective to periodontal disease and smoking is a risk factor. Previously it was considered that HIV-infected patients had an increased risk of developing. However, these findings have been contested and, HAART has been considered as a protector against periodontal disease^29^. Interestingly, our results showed a correlation between the duration of treatment and not with a specific type of therapy (HAART, mono, or dual therapy). It is important to highlight that periodontal disease is related to plaque and pooh hygiene as with non-HIV-infected individuals^30^, which were not evaluated in this study.

Unlike conventional gingivitis, LGE usually does not respond to routine oral hygiene measures^15^. This lesion is considered one of the most common periodontal diseases in HIV-infected individuals, and etiology could be related to invasion by *Candida* species in the subgingival tissue^29^, ^31^. Because of that, some studies consider that LGE should be considered another variant type from candidiasis and not a distinct disease in the HIV-infected patients^14^. Although this condition is rarely found in patients undergoing HAART^7^, ^13^, ^14^, in our study the prevalence was considerable (10 of 105 patients) and should be considered by clinicians during the examination of patients.

*Candida* is a common opportunistic pathogen and Oral Candidiasis (OC) in the variant of pseudomembranous candidiasis was seen in 14 patients using ART. These values are corroborated with the data found in the literature^5^, ^7^, ^24^, ^32^. Also, in our study, the OC was observed in 7 patients that presented a low CD4/CD8 ratio (<1), which indicates an important finding to clinicians. OHL is associated with Epstein-Barr virus infection in epithelial cells and immunosuppressed patients are most affected^33^. In this study, the prevalence of OHL is low, which is like the results of previous studies^5^, ^7^, ^9^, ^27^. El Howati et al.^12^ confirms these findings in a systematic review, which shows that OHL was significantly less in groups receiving ART compared with those not on treatment.

It is important to highlight that although there was a decrease in associated oral manifestations with HIV patients after ART therapy, the prolonged use of some therapies can increase some types of oral lesions, such as hyperpigmentation, xerostomia, and hypertrophy of salivary glands^6^. In our study, 14.29% of the patients had oral hyperpigmentation, these data also have been found in other studies^7^, ^12^, ^20^, ^32^. The incidence of hyperpigmentation has been attributed to taking drugs during ART. The zidovudine is considered one of the drugs of the ART that can induce melanotic changes in the oral mucosa^25^, ^33^. However, in this study oral hyperpigmentation was related to smoking than type and duration of treatment.

This study had some limitations such as only patients over 18 years of age participated in the study and the study group was restricted to the Governador Valadares region, not the entire state, or country. Not all patients had CD4 + T cell counts and viral loads data at the time of oral examination. Also, in this study, only dental mobility and gingivitis were evaluated due to structural limitations.

These results demonstrated a presence of associated oral manifestations in patients on ART therapy, although this incidence is high only for periodontal disease with or without mobility. However, it must be important to emphasize that the presence or absence of oral lesions can serve as an indicator of HIV and indexes of treatment success^14^.

## Conclusion

Our results showed that among patients living with HIV undergoing antiretroviral treatment oral lesions can be observed, predominantly periodontal disease with or without dental mobility. Pseudomembranous candidiasis and oral hairy leukoplakia were also observed, however less commonly. No relationship was found between associated oral manifestations in HIV patients and the start of the treatment, TCD4+ and TCD8+ cell count, TCD4:TCD8 ratio, or viral load. The data indicate that there is a protective effect of duration of treatment with relation to periodontal disease with mobility and that hyperpigmentation seems to be more related to smoking than type and duration of treatment.

## Data availability statement

The data that support the findings of this study are available on request from the corresponding author. The data are not publicly available due to privacy or ethical restrictions.

## Funding

This research did not receive any specific grant from funding agencies in the public, commercial, or not-for-profit sectors.

## Ethics approval statement

The study protocol was approved by the research committee at the Federal University of Juiz de Fora, Brazil (#1.821.072).

## Patient consent statement

Subjects were given both verbal and written information about the nature of the study and written consent was obtained.

## Conflicts of interest

The authors declare no conflicts of interest.
